# Explainable machine learning for neurological outcome prediction in out-of-hospital cardiac arrest survivors undergoing targeted temperature management: a multi-cohort validation study

**DOI:** 10.1186/s12984-025-01821-7

**Published:** 2025-12-01

**Authors:** Oluwaseun Adebayo Bamodu, Yu-Xin Goh, Chien-Tai Hong, Po-Chih Chen, Wei-Ting Chiu, Lung Chan, Chen-Chih Chung

**Affiliations:** 1 Department of Prevention and Community Health, Milken Institute School of Public Health, The George Washington University, Washington, D.C USA; 2 Directorate of Postgraduate Studies, School of Clinical Medicine, Muhimbili University of Health and Allied Sciences, Ilala District, Dar es Salaam, Tanzania; 3 Department of Neurology, Taipei Medical University—Shuang Ho Hospital, New Taipei City, Taiwan; 4 Department of Neurology, School of Medicine, College of Medicine, Taipei Medical University, 250 Wuxing Street, Xinyi District, Taipei, Taiwan; 5 Division of Critical Care Medicine, Department of Emergency and Critical Care Medicine, Taipei Medical University—Shuang Ho Hospital, New Taipei City, Taiwan

**Keywords:** Explainable AI, Interpretability, Machine learning, Neurological prediction, Out-of-hospital cardiac arrest, Risk stratification

## Abstract

**Background:**

Early risk assessment in comatose survivors of out-of-hospital cardiac arrest (OHCA) remains clinically challenging, particularly for patients undergoing targeted temperature management (TTM). This study aimed to develop and externally validate an interpretable machine learning model to predict neurological outcomes in TTM-treated comatose OHCA survivors, leveraging multinational registry data to improve generalizability and early-phase characterization.

**Methods:**

Data were derived from two multi-center registries: the Korean Hypothermia Network prospective registry (KORHN-pro, *n* = 1,050) and the Taiwan Network of Targeted Temperature Management for Cardiac Arrest (TIMECARD, *n* = 393). Adult OHCA patients who remained comatose after return of spontaneous circulation (ROSC) were included. The KORHN-pro dataset was used for model development and internal validation via 10-fold cross-validation, while the TIMECARD registry served as an independent external validation cohort. The primary outcome was a favorable neurological status (Cerebral Performance Category score 1–2) at hospital discharge. Eighteen pre-intervention variables were used to train seven machine learning algorithms. The best-performing model was selected based on discrimination metrics. Model interpretability was evaluated using Shapley Additive exPlanations (SHAP) to examine feature importance, interaction effects, and case-level predictions.

**Results:**

The eXtreme Gradient Boosting algorithm achieved the highest performance, with an area under the receiver operating characteristic curve of 0.925 in internal and 0.852 in external validation. Key predictive determinants included initial shockable rhythm, time to ROSC, adrenaline dose, and Glasgow Coma Scale motor score. SHAP analysis highlighted synergistic effects among features, particularly between cardiac rhythm and early neurological status, which were further illustrated through case-level explanations in the external validation cohort.

**Conclusion:**

This study presents an interpretable machine learning model for early neurological stratification in comatose OHCA survivors undergoing TTM. Using multinational registry data, the model demonstrated robust performance across both development and external validation cohorts. By integrating clinically relevant predictors, this approach provides individualized estimates to support early assessment and guide future therapeutic considerations. Notably, this study was not designed to compare different TTM temperature strategies, and results should not be interpreted in that context. The explainable framework is intended to complement clinical evaluation without replacing physician judgment or informing treatment withdrawal decisions.

**Supplementary Information:**

The online version contains supplementary material available at 10.1186/s12984-025-01821-7.

## Introduction

Out-of-hospital cardiac arrest (OHCA) represents a global public health challenge, with persistently high mortality and neurological morbidity despite advances in post-resuscitation care [1, 2]. For comatose survivors following the return of spontaneous circulation (ROSC), early risk stratification is critical for guiding treatment intensity and resource allocation [3]. However, heterogeneous patient populations and variability in post-arrest care across healthcare systems emphasize the need for robust, generalizable predictions.

Targeted temperature management (TTM) is a cornerstone of post-cardiac arrest care, aimed at mitigating ischemic brain injury through controlled hypothermia [4–6]. While TTM demonstrates neurological benefits, particularly in patients with shockable rhythms, its overall efficacy continues to be debated due to inconsistencies in patient selection, protocol execution, and outcome assessment [7–9]. Despite extensive prior research, key prognostic uncertainties persist, underscoring the need for individualized prediction models to guide early risk stratification.

Existing prognostic tools, including the OHCA and Cardiac Arrest Hospital Prognosis (CAHP) scores, offer practical frameworks but are constrained by linear assumptions and limited capacity to capture complex physiological interactions under TTM protocols [10–12]. This restricts their application in personalized neuroprotective strategies where non-linear and synergistic effects are critical.

Recent advances in machine learning have introduced opportunities for more nuanced prediction of outcomes by using high-dimensional data and capturing non-linear relationships among clinical variables [13, 14]. However, model opacity remains a barrier to clinical adoption, as interpretability is crucial for integration into critical care decision-making [15–20].

To address these limitations, we hypothesized that routinely available baseline clinical variables encode prognostic information that can be elucidated through an explainable machine learning framework. Our primary objective was to develop and externally validate an interpretable prediction model for neurological outcomes among comatose OHCA survivors treated with TTM. Leveraging prospectively collected multi-center registries from Korea and Taiwan, we trained and compared multiple machine learning algorithms using 18 clinical variables available at ROSC. Secondary objectives included benchmarking algorithm performance and exploring feature importance through Shapley Additive exPlanations (SHAP) to illustrate non-linear effects, interactions, and patient-specific predictions. This study assesses the potential of explainable artificial intelligence (AI) to enhance early neuroprotective decision-making and individualized care planning in post-arrest management.

## Materials and methods

### Data sources

This retrospective cohort study utilized two independently maintained, prospectively collected clinical registries: the Korean Hypothermia Network prospective registry (KORHN-pro) and the Taiwan Network of Targeted Temperature Management for Cardiac Arrest (TIMECARD).

### Participants

The KORHN-pro registry is a web-based, prospective, multi-center database initiated by a clinical research consortium of 22 academic hospitals across South Korea, enrolling adults with sustained ROSC after OHCA who remained comatose on admission between October 2015 and December 2018. Variables were collected using standardized forms based on the Utstein Resuscitation Registry template [21–23]. This dataset was used for model development and internal validation.

The TIMECARD registry is a nationwide, multi-center observational initiative launched by the Taiwan Society of Emergency and Critical Care Medicine, involving ten tertiary medical centers between January 2014 and September 2019. Standardized electronic case report forms were used to systematically collect data on demographics, medical history, arrest characteristics, prehospital and in-hospital interventions, complications, and outcomes [17, 24]. This cohort served as an independent external validation dataset.

All data were de-identified and collected under institutional review board–approved protocols. Variable definitions and data structures were harmonized to ensure consistency. All patients in both registries received TTM as part of post-resuscitation care [21–24]. Only baseline variables available before the initiation of post-ROSC interventions were used for model development and evaluation.

### TTM protocol

TTM was implemented in Korea and Taiwan according to institutional protocols aligned with regional and international postcardiac arrest care guidelines. In Korea, TTM was conducted under the KORHN-pro registry protocol, targeting 33 °C for 24 h followed by active rewarming, typically at 0.25 °C per hour in most patients (89.7%) [21, 22]. In Taiwan, participating centers implemented a standardized protocol with target temperatures ranging from 32 to 36 °C, maintained for 24 h, and followed by controlled rewarming at 0.25–0.5 °C per hour to prevent rebound hyperthermia [25]. Notably, patients were not stratified by specific temperature targets; instead, this analysis focused on predicting neurological outcomes across the entire TTM-treated population.

Withdrawal of life-sustaining therapy (WLST) practices differed between the two registries. In the KORHN-pro registry, WLST events were systematically documented, with an incidence of approximately 2.7% [23]. In contrast, the TIMECARD registry did not systematically collect WLST data, and the available information was insufficient for reliable estimation within the cohort [24, 26].

### Inclusion and exclusion criteria

Data from the two registries were integrated using harmonized eligibility criteria to ensure comparability.

Patients were eligible for inclusion if they:

(i) were aged ≥ 18 years;
(ii) experienced OHCA;
(iii) achieved return of spontaneous circulation (ROSC) following cardiopulmonary resuscitation (CPR);
(iv) remained comatose after ROSC, defined as a Glasgow Coma Scale (GCS) < 8 or inability to follow commands; and.
(v) received TTM within 12 h of ROSC.

Exclusion criteria included:

(i) impaired consciousness before arrest, defined as a pre-arrest Cerebral Performance Category (CPC) ≥ 3;
(ii) history of cerebrovascular accident (CVA);
(iii) other significant pre-existing neurological disorders;
(iv) traumatic etiology of arrest;
(v) missing CPC score at discharge; and
(vi) terminal illness with a life expectancy < 6 months.

Although the TIMECARD registry initially excluded patients with pre-arrest CPC ≥ 3, this was not an explicit criterion in the KORHN-pro protocol. The same neurological exclusion criteria were retrospectively applied to both datasets during preprocessing to ensure consistency.

From the KORHN-pro registry, 1190 patients were initially screened. After excluding cases with traumatic arrest (*n* = 21), CVA (*n* = 58), other neurological disorders (*n* = 56), and missing CPC score (*n* = 5), a total of 1050 patients comprised the development cohort. From the TIMECARD registry, 580 patients were screened. After excluding in-hospital cardiac arrests (*n* = 111), traumatic arrests (*n* = 10), CVA (*n* = 65), and one case with missing ROSC time, 393 patients were included in the external validation cohort.

**Fig. 1:**
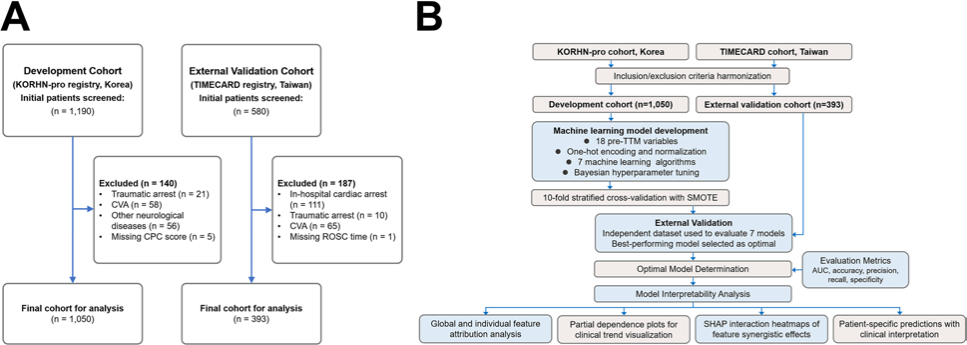
Study cohorts and modelling workflow. **A** patient selection from two national registries (KORHN-pro and TIMECARD), with applied exclusion criteria and final cohort sizes. **B** machine learning framework for early neurological risk stratification in comatose patients following OHCA. Abbreviations: AUC, area under the receiver operating characteristic curve; CVA, cerebrovascular accident; IHCA, in-hospital cardiac arrest; KORHN-pro, Korean Hypothermia Network prospective registry; OHCA, out-of-hospital cardiac arrest; SHAP, Shapley Additive exPlanations; SMOTE, Synthetic Minority Oversampling Technique; TIMECARD, Taiwan network of Targeted Temperature Management for Cardiac arrest.

The patient selection process is summarized in Fig. 1A.

### Statistical analyses

Continuous variables were tested for normality using the Shapiro–Wilk test. Normally distributed variables were presented as mean ± standard deviation and compared with the independent t-test. Non-normally distributed variables were summarized as median (interquartile range, IQR) and compared with the Mann–Whitney U test.

Categorical variables were summarized as frequencies and percentages and compared using the chi-square test or Fisher’s exact test, as appropriate. Two-tailed *p*-values < 0.05 were considered statistically significant. Analyses were performed using STATISTICA (version 14.0; TIBCO Software Inc., Tulsa, OK, USA).

### Outcomes

The primary outcome was neurological status at hospital discharge, measured with the CPC scale [22, 25]. Favorable outcome was defined as CPC 1–2 (good cerebral function or moderate disability), and unfavorable outcome as CPC 3–5 (severe disability, coma, vegetative state, or death).

In KORHN-pro, bedside CPC scoring was performed by treating physicians, with final categorization based on clinical consensus. In TIMECARD, trained research personnel assigned CPC scores using structured chart review, incorporating discharge documentation and neurological assessments. Inter-rater reliability procedures were implemented to ensure consistency.

In-hospital mortality (CPC = 5) was included in descriptive analyses but not modeled as a separate endpoint. As the TIMECARD registry lacked post-discharge follow-up, outcome assessment was restricted to discharge status.

### Model development

Analyses were performed in Python (version 3.10.9; Python Software Foundation, Wilmington, DE, USA). Seven machine learning algorithms were evaluated: logistic regression, support vector machines, random forest, extremely randomized trees, light gradient boosting machines (LightGBM), artificial neural networks, and eXtreme Gradient Boosting (XGBoost).

Model development was based on the KORHN-pro cohort. Eighteen clinical variables available at the time of ROSC and uniformly collected across registries were used as predictors **(**Table 1**)**. The GCS motor score was the only variable requiring subjective interpretation, assessed by treating physicians or nursing staff during routine care. No additional feature selection procedures were applied.

**Table 1:** Baseline clinical characteristics of the development cohort by neurological outcome at hospital discharge

Variables	Whole cohort	Outcomes at discharge			
		Favorable	Unfavorable	OR (95% CI)	*p*-value
Number of patients	1050 (100)	340 (32.4)	710 (67.6)		
Clinical predictors					
Age, years, median (IQR)	57 (46–69)	53.0 (43.25–60)	60 (48–72)	0.97 (0.96–0.98)	< 0.0001***
Female sex, n (%)	301 (28.7)	69 (20.3)	232 (32.7)	1.91 (1.40–2.59)	< 0.0001***
Witnessed collapse, n (%)	739 (70.4)	292 (85.9)	447 (63.0)	3.58 (2.54–5.03)	< 0.0001***
Bystander CPR, n (%)	659 (62.8)	238 (70.0)	421 (59.3)	1.60 (1.22–2.11)	0.0007***
First monitored rhythm				11.20 (8.26–15.19)	< 0.0001***
Shockable, n (%)	405 (38.6)	255 (75.0)	150 (21.1)		
Non-shockable, n (%)	615 (58.6)	70 (20.6)	545 (76.8)		
Coronary artery disease, n (%)	136 (13.0)	58 (17.1)	78 (11.0)	1.67 (1.15–2.41)	0.0078**
Arrhythmia, n (%)	59 (5.6)	18 (5.3)	41 (5.8)	0.91 (0.52–1.61)	0.8862
Heart failure, n (%)	49 (4.7)	14 (4.1)	35 (4.9)	0.83 (0.44–1.56)	0.6405
Hypertension, n (%)	352 (33.5)	92 (27.1)	260 (36.6)	0.64 (0.48–0.85)	0.0021**
Diabetes mellitus, n (%)	233 (22.2)	44 (12.9)	189 (26.6)	0.41 (0.29–0.59)	< 0.0001***
Pulmonary disease, n (%)	77 (7.3)	12 (3.5)	65 (9.2)	0.36 (0.19–0.68)	0.0027**
Malignancy, n (%)	60 (5.7)	15 (4.4)	45 (6.3)	0.68 (0.37–1.24)	0.2557
Chronic kidney disease, n (%)	72 (6.9)	9 (2.6)	63 (8.9)	0.28 (0.14–0.57)	0.0001***
Liver cirrhosis, n (%)	18 (1.7)	1 (0.3)	17 (2.4)	0.12 (0.02–0.91)	0.0108*
Time to ROSC, min, median (IQR)	29 (17–43)	18.0 (12–26)	35 (23–48)	0.94 (0.93–0.95)	< 0.0001***
Adrenaline dose during CPR, mg, median (IQR)	2 (0–3)	0 (0–1)	2 (1–4)	0.64 (0.58–0.70)	< 0.0001***
Arrest etiology				6.20 (4.34–8.84)	< 0.0001***
Cardiac, n (%)	677 (64.5)	298 (87.6)	379 (53.4)		
Non-cardiac, n (%)	373 (35.5)	42 (12.4)	331 (46.6)		
GCS motor score, median (IQR)	1 (1–2)	2 (1–4)	1 (1–1)	2.35 (2.07–2.67)	< 0.0001***
Outcomes at hospital discharge					
CPC at hospital discharge, n (%)				NA†	< 0.0001***
1	295 (28.1)	295 (86.8)	0 (0)		
2	45 (4.3)	45 (13.2)	0 (0)		
3	37 (3.5)	0 (0)	37 (5.2)		
4	190 (18.1)	0 (0)	190 (26.8)		
5	483 (46.0)	0 (0)	483 (68.0)		
Survival to hospital discharge, n (%)	567 (54.0)	340 (100.0)	227 (32.0)	NA†	< 0.0001***

Favorable neurological outcomes were defined as CPC scores of 1–2 and unfavorable outcomes as CPC scores of 3–5

Continuous variables are presented as median (IQR), and categorical variables as counts (percentages). Percentages are calculated based on the total number of patients in each group

†Variables under “Outcomes at hospital discharge” (CPC at discharge and Survival to hospital discharge) were excluded from regression analyses because of their collinearity with the primary outcome. They are reported descriptively, and ORs were not estimable. All p-values correspond to comparisons between favorable and unfavorable outcome groups. *p < 0.05; **p < 0.01; ***p < 0.001

CI, confidence interval; CPC, Cerebral Performance Category; CPR, cardiopulmonary resuscitation; GCS, Glasgow Coma Scale; IQR, interquartile range; OR, odds ratio; ROSC, return of spontaneous circulation

### Preprocessing

All preprocessing steps were conducted independently within each dataset to preserve data integrity and avoid leakage. Missing values were handled using non-iterative methods, with continuous variables imputed using the median and categorical variables using the most frequent category within each cohort. For the external validation cohort (TIMECARD), imputation values were derived exclusively from within the same dataset to maintain statistical independence from the development cohort (KORHN-pro).

Categorical variables were one-hot encoded. Continuous variables were standardized using z-score normalization (mean = 0, standard deviation = 1) for algorithms sensitive to input scaling, including logistic regression, support vector machines, and artificial neural networks. Tree-based models (random forest, extremely randomized trees, LightGBM, and XGBoost) were trained on unscaled data, leveraging their inherent scale invariance.

A favorable neurological outcome (CPC 1–2) was defined as the positive class for classification and metric calculation for all models.

### Hyperparameter configuration

Hyperparameter tuning was performed independently for all seven machine learning models using Optuna Bayesian optimization with 10-fold cross-validation. The mean F1 score across folds served as the optimization objective to identify the best hyperparameter configuration. To ensure robustness in hyperparameter selection and achieve stable convergence of the primary performance metric, we employed 30,000 Bayesian optimization trials. This number was determined empirically to minimize stochastic variation during tuning and to improve consistency in parameter selection for final model deployment. Bayesian optimization constructs a probabilistic surrogate model of the objective function, efficiently exploring the hyperparameter space and identifying promising configurations [27, 28]. Separately, within each fold, the classification threshold was further adjusted to maximize the F1 score on the validation partition, ensuring an optimal balance between precision and recall at the decision level.

To address class imbalance in the training dataset, the Synthetic Minority Oversampling Technique (SMOTE) was applied exclusively to the training partitions within each cross-validation fold, while validation folds and the external test cohort were left unaltered. This ensured that resampling was strictly confined to the training data, thereby minimizing the risk of information leakage [18, 29]. The same hyperparameter tuning and resampling procedure was uniformly applied across all algorithms.

### External validation

External validation was conducted on an independent cohort from the TIMECARD registry (Table 2). Each of the 10 models from internal cross-validation was deployed separately to the external dataset without retraining. For each patient, predicted probabilities were generated using the predict_proba method (XGBoost v2.1.3), and the outputs of all 10 models were averaged to obtain a final ensemble risk score.

**Table 2:** Baseline clinical characteristics of the external validation cohort by neurological outcome at hospital discharge

Variables	Whole cohort	Outcomes at discharge			
		Favorable	Unfavorable	OR (95% CI)	*p*-value
Number of patients, n (%)	393 (100)	91 (23.2)	302 (76.8)		
Clinical predictors					
Age, years, median (IQR)	64 (52.6–74.7)	56.7 (42.4–66.3)	65.7 (54.7–76.5)	0.96 (0.95–0.98)	< 0.0001***
Female sex, n (%)	138 (35.1)	23 (25.3)	115 (38.1)	1.82 (1.07–3.08)	0.0248*
Witnessed collapse, n (%)	305 (77.6)	81 (89.0)	224 (74.2)	2.82 (1.39–5.71)	0.0025**
Bystander CPR, n (%)	247 (62.8)	69 (75.8)	178 (58.9)	2.18 (1.28–3.72)	0.0042**
First monitored rhythm				6.54 (3.84–11.13)	< 0.0001***
Shockable, n (%)	162 (41.2)	68 (74.7)	94 (31.1)		
Non-shockable, n (%)	231 (58.8)	23 (25.3)	208 (68.9)		
Coronary artery disease, n (%)	107 (27.2)	24 (26.4)	83 (27.5)	0.95 (0.56–1.61)	0.8936
Arrhythmia, n (%)	38 (9.7)	9 (9.9)	29 (9.6)	1.03 (0.47–2.27)	1.0000
Heart failure, n (%)	56 (14.2)	8 (8.8)	48 (15.9)	0.51 (0.23–1.12)	0.1223
Hypertension, n (%)	208 (52.9)	50 (54.9)	158 (52.3)	1.11 (0.69–1.78)	0.7191
Diabetes mellitus, n (%)	146 (37.2)	24 (26.4)	122 (40.4)	0.53 (0.31–0.89)	0.0184*
Pulmonary disease, n (%)	37 (9.4)	5 (5.5)	32 (10.6)	0.49 (0.19–1.30)	0.2173
Malignancy, n (%)	37 (9.4)	3 (3.3)	34 (11.3)	0.27 (0.08–0.90)	0.0232*
Chronic kidney disease, n (%)	53 (13.5)	7 (7.7)	46 (15.2)	0.46 (0.20–1.07)	0.0793
Liver cirrhosis, n (%)	11 (2.8)	1 (1.1)	10 (3.3)	0.32 (0.04–2.57)	0.4690
Time to ROSC, min, median (IQR)	32 (20–44)	24 (15–34)	34.5 (25–45.75)		< 0.0001***
Adrenaline dose during CPR, mg, median (IQR)	1 (0–5)	0 (0–3)	3 (0–6)	0.78 (0.70–0.86)	< 0.0001***
Arrest etiology				2.84 (1.47–5.47)	0.0010***
Cardiac, n (%)	290 (73.8)	79 (86.8)	211 (69.9)		
Non-cardiac, n (%)	103 (26.2)	12 (13.2)	91(30.1)		
GCS motor score, median (IQR)	1 (1–1)	1 (1–3)	1 (1–1)	2.04 (1.63–2.56)	< 0.0001***
Outcome at hospital discharge					
CPC at hospital discharge, n (%)				NA†	< 0.0001***
1	72 (18.3)	72 (79.1)	0 (0)		
2	19 (4.8)	19 (20.9)	0 (0)		
3	23 (5.9)	0 (0)	23 (7.6)		
4	54 (13.7)	0 (0)	54 (17.9)		
5	225 (57.3)	0 (0)	225 (74.5)		
Survival to hospital discharge, n (%)	168 (42.7)	91 (100.0)	77 (25.5)	NA†	< 0.0001***

Favorable neurological outcomes were defined as CPC scores of 1–2 and unfavorable outcomes as CPC scores of 3–5

Continuous variables are presented as median (IQR), and categorical variables as counts (percentages). Percentages are calculated based on the total number of patients in each group

†Variables under “Outcomes at hospital discharge” (CPC at discharge and Survival to hospital discharge) were excluded from regression analyses because of their collinearity with the primary outcome. They are reported descriptively, and ORs were not estimable. All p-values correspond to comparisons between favorable and unfavorable outcome groups. *p < 0.05; **p < 0.01; ***p < 0.001

CI, confidence interval; CPC, Cerebral Performance Category; CPR, cardiopulmonary resuscitation; GCS, Glasgow Coma Scale; IQR, interquartile range; OR, odds ratio; ROSC, return of spontaneous circulation

The classification threshold, determined by optimizing the F1 score during internal validation, was fixed prior to testing and not recalibrated for the external cohort.

Model performance was quantified using standard metrics: area under the receiver operating characteristic curve (AUC), accuracy, precision, recall (sensitivity), and specificity. Among the evaluated algorithms, the one with the highest AUC on the external dataset was selected for interpretation. To assess the reliability of predicted probabilities, calibration was examined using calibration curves and the Brier score [30].

### Interpretability and explainability analysis

To enhance model transparency, we used SHAP to explain the final model’s predictions and visualize feature contributions. SHAP values quantify the direction and magnitude of each feature’s contribution to a given prediction by allocating the predicted probability across input variables, effectively decomposing the model output into interpretable components [31].

Global feature importance was visualized using SHAP summary plots, partial dependence plots (PDPs) were used to capture non-linear associations, and interaction heatmaps highlighted synergistic effects. Case-level explanations were included to illustrate how key predictors shaped individualized risk estimates [31, 32]. Figure 1B illustrates the analytical pipeline for model training, validation, and evaluation.

This study was reported in accordance with the TRIPOD–AI (Transparent Reporting of a multivariable prediction model for Individual Prognosis Or Diagnosis–Artificial Intelligence extension) guideline [33]; the completed checklist is provided in Supplementary Table 1.

## Results

### Cohort characteristics

In the KORHN-pro registry, 1,190 OHCA patients were initially screened. After excluding patients with traumatic arrest (*n* = 21), CVA (*n* = 58), other major neurological disorders (*n* = 56), and missing outcome data (*n* = 5), a total of 1,050 patients were included for analysis (Fig. 1A).

In the TIMECARD registry, 580 patients were screened. Exclusions comprised in-hospital cardiac arrests (*n* = 111), traumatic arrests (*n* = 10), CVA (*n* = 65), and one case with missing ROSC time, yielding 393 patients for external validation.

### Development cohort (KORHN-pro)

The development cohort comprised 1050 adult comatose survivors of OHCA enrolled in the Korean KORHN-pro registry. Favorable neurological outcome (CPC 1–2) was observed in 340 patients (32.4%), while 710 (67.6%) had unfavorable outcomes (CPC 3–5). The overall survival rate for hospital discharge was 54.0%. Patients with favorable outcomes were generally younger, more likely to have witnessed arrests with bystander-performed CPR, and more frequently presented with initial shockable rhythms. They also exhibited shorter time to ROSC, lower adrenaline doses administered during resuscitation, and higher GCS motor scores at admission. Cardiac etiology was more common in this group, whereas diabetes mellitus, hypertension, chronic kidney disease, and pulmonary disease were more prevalent among patients with unfavorable outcomes (Table 1**)**.

### External validation cohort (TIMECARD)

The external validation cohort included 393 comatose OHCA patients from the Taiwan TIMECARD registry during a period overlapping with the development cohort. Of these, 91 patients (23.2%) achieved favorable outcomes, and 302 (76.8%) had poor outcomes. Patients with favorable outcomes tended to be younger, with more frequent witnessed arrests, bystander-performed CPR, and initial shockable rhythms. They also exhibited shorter ROSC times, lower cumulative adrenaline use, and higher GCS motor scores at admission. Cardiac etiology predominated in the favorable outcome group, while comorbidities, including diabetes mellitus and malignancy, were more prevalent in the unfavorable outcome group (Table 2**)**.

### Performance of machine learning models

**Fig. 2:**
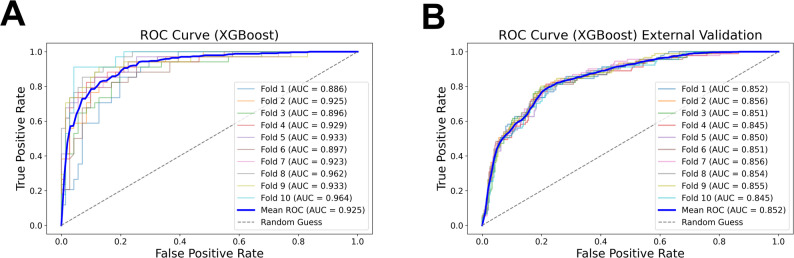
Receiver operating characteristic (ROC) curves of the XGBoost model. **A** internal validation ROC curves derived from 10-fold cross-validation on the development cohort. Individual fold curves are displayed with their respective AUC values. The bold blue line represents the mean ROC curve. **B** external validation ROC curves generated by applying the 10 trained models to the independent validation cohort. The bold blue line indicates the mean ROC curve. The dashed gray line in both panels represents the performance of random classification. Abbreviations: AUC, area under the receiver operating characteristic curve; ROC, receiver operating characteristic; XGBoost, eXtreme gradient boosting.

In the development cohort (KORHN-pro), all seven machine learning algorithms demonstrated adequate performance, with the XGBoost model achieving the highest discriminative ability. In 10-fold cross-validation, XGBoost yielded a mean AUC of 0.925 (95% CI 0.91–0.94) (Fig. 2A) for predicting favorable neurological outcomes, with corresponding accuracy of 0.863 (95% CI 0.84–0.89), precision of 0.770 (95% CI 0.72–0.82), sensitivity of 0.835 (95% CI 0.81–0.86), specificity of 0.876 (95% CI 0.84–0.91).

On external validation with the independent TIMECARD cohort, the model yielded an AUC of 0.852, accuracy of 0.809, precision of 0.584, sensitivity of 0.617, and specificity of 0.867, without additional retraining (Fig. 2B).

Comparative performance metrics for all algorithms are summarized in Supplementary Tables 2 and 3. For the final XGBoost model, 30,000 Bayesian optimization trials were conducted to explore the hyperparameter space. Corresponding hyperparameter values, search ranges, and SMOTE handling are provided in Supplementary Table 4.

Model calibration was assessed using a calibration curve and Brier score. Predicted probabilities showed reasonable agreement with observed outcomes on external validation (Brier score = 0.129; Supplementary Figure) [30].

To further evaluate clinical utility, we examined patients with a low predicted probability of favorable outcomes. Among 90 patients (22.9%) with an estimated probability below 5% as predicted by the XGBoost model, only one achieved favorable recovery, corresponding to a negative predictive value (NPV) of 98.9%.

**Fig. 3:**
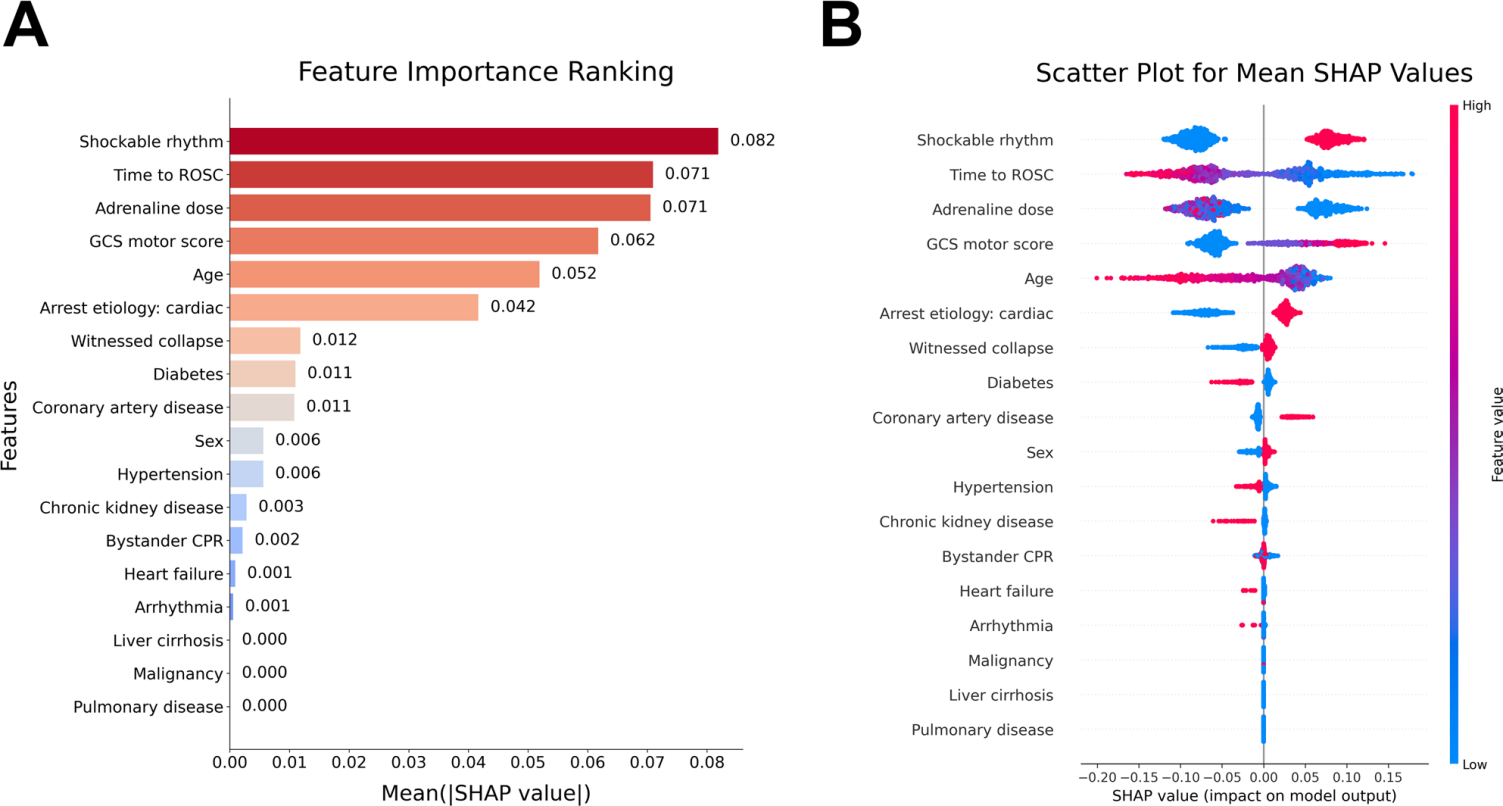
SHAP-based feature importance and summary plots of the XGBoost model. **A** bar plot depicting all features’ mean absolute SHAP values, hierarchically arranged by their contribution to neurological outcome prediction. Higher mean SHAP values correspond to a more decisive influence on model predictions. **B** SHAP summary plot demonstrating both magnitude and directionality of feature effects on model output. Individual points represent single patient observations, with SHAP values on the x-axis. The color gradient indicates feature value intensity (blue: lower values; red: higher values). Features are arranged vertically in descending order of importance. Abbreviations: CPR, cardiopulmonary resuscitation; CAD, coronary artery disease; GCS, Glasgow Coma Scale; ROSC, return of spontaneous circulation; SHAP, Shapley Additive exPlanations; XGBoost, eXtreme gradient boosting.

### Hierarchical ranking and visual interpretation of prognostic indicators

SHAP analysis quantified the hierarchical contribution of predictors in the XGBoost model. Feature importance, quantified by mean absolute SHAP values, identified initial shockable rhythm, shorter time to ROSC, lower cumulative adrenaline doses, higher GCS motor scores, and younger age as the most influential features (Fig. 3A).

The SHAP summary plot delineates the directional contribution of individual features (Fig. 3B). Shockable rhythm and higher GCS motor scores (depicted in red) exhibited positive values, indicating association with favorable outcomes. Conversely, advanced age, prolonged time to ROSC, and higher adrenaline doses generated negative SHAP values, predicting unfavorable outcomes. Diabetes mellitus modestly contributed to unfavorable predictions, while coronary artery disease showed a positive association, potentially reflecting the influence of identified cardiac etiology.

**Fig. 4:**
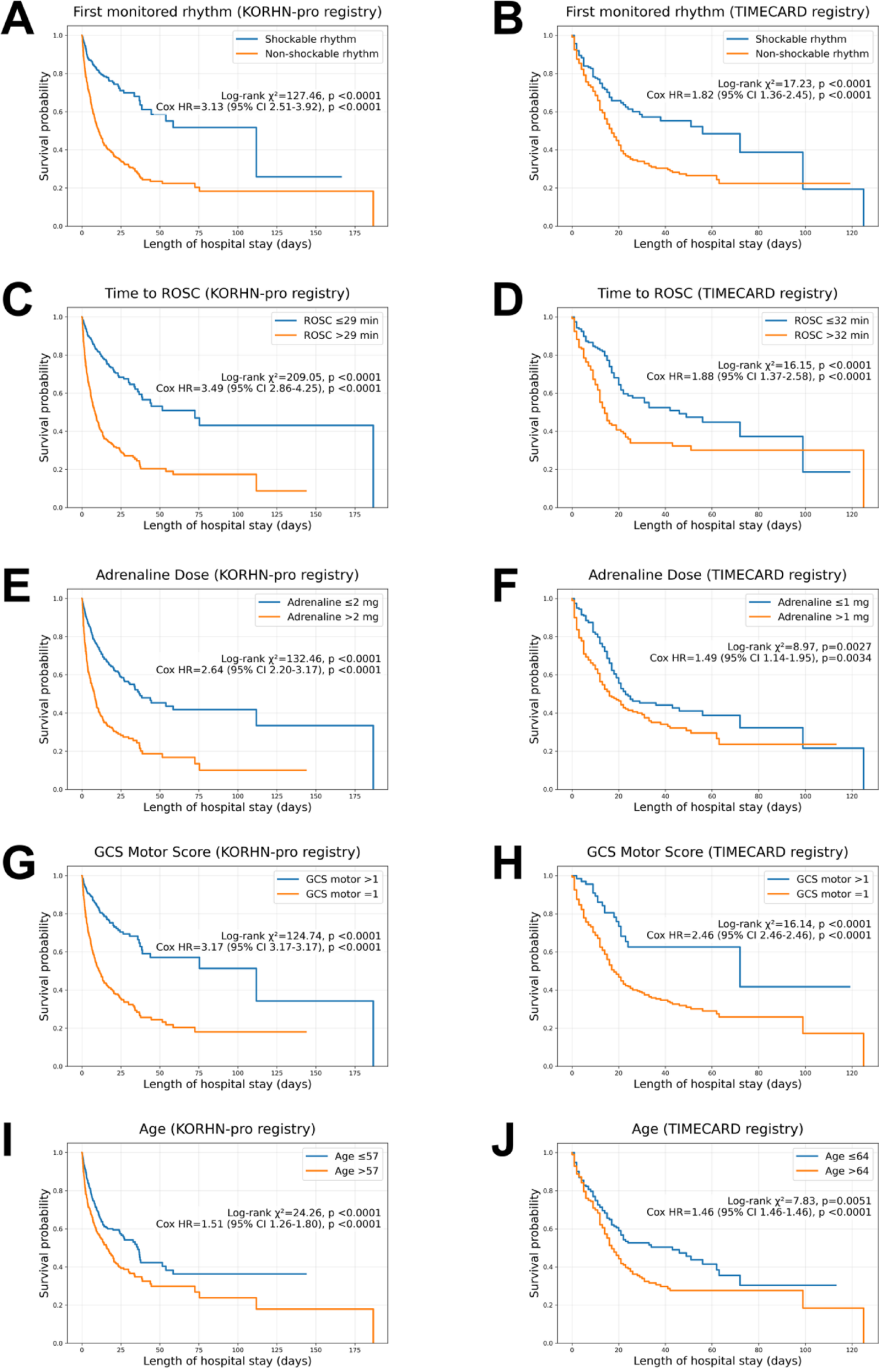
Kaplan–Meier in-hospital survival curves by clinical variables in development (KORHN-pro) and validation (TIMECARD) cohorts. Outcomes were stratified by (**A–B**) initial rhythm, **C–D** time to ROSC, **E–F** adrenaline dose, **G–H** GCS motor score, and **I–J** age. Shockable rhythm, shorter time to ROSC, lower adrenaline dose, higher GCS motor scores, and younger age were associated with improved survival in both the development and validation cohorts. Group differences were assessed with the log-rank test, and hazard ratios (HR) with 95% confidence intervals were estimated using Cox regression. Continuous variables were dichotomized at cohort-specific medians. X-axis differs by cohort due to maximum follow-up duration. Abbreviations:GCS, Glasgow Coma Scale; HR, hazard ratio; ROSC, return of spontaneous circulation.

The prognostic relevance of these machine learning–derived features was further corroborated by conventional statistical analyses. Kaplan-Meier survival curves stratified by initial rhythm, time to ROSC, adrenaline dose, GCS motor score, and age demonstrated significant survival differences across both cohorts (Fig. 4). In multivariable logistic regression (Table 3), shockable rhythm, lower adrenaline dose, higher GCS motor score, and younger age were independently associated with favorable neurological outcomes, while time to ROSC demonstrated a strong association in the development cohort.

**Table 3:** Multivariable logistic regression for favorable neurological outcome at hospital discharge (per-unit increase of predictors)

Variable	Adjusted OR	95% CI	*p*-value
Development cohort (KORHN-pro)			
Shockable	9.980	6.860–14.520	< 0.0001
Time to ROSC (per 1 min)	0.956	0.942–0.970	< 0.0001
Adrenaline dose (per 1 mg)	0.917	0.841–0.999	0.0473
GCS motor score (per 1 point)	1.818	1.557–2.123	< 0.0001
Age (per 1 year)	0.967	0.955–0.979	< 0.0001
External validation cohort (TIMECARD)			
Shockable	6.558	3.433–12.529	< 0.0001
Time to ROSC (per 1 min)	1.000	0.998–1.002	0.8842
Adrenaline dose (per 1 mg)	0.783	0.695–0.881	< 0.0001
GCS motor score (per 1 point)	1.432	1.081–1.895	0.0122
Age (per 1 year)	0.976	0.957–0.996	0.0166

Logistic regression models were constructed separately for the development cohort (KORHN-pro, *n* = 1050) and the external validation cohort (TIMECARD, *n* = 393). A favorable neurological outcome was defined as a Cerebral Performance Category of 1–2 at hospital discharge. Shockable rhythm was coded as binary (ventricular fibrillation or pulseless ventricular tachycardia vs. non-shockable). Other predictors were modeled as continuous variables: time to ROSC (per 1 min), adrenaline dose (per 1 mg), GCS motor score (per 1 point), and age (per 1 year). Odds ratios greater than 1 indicate a higher likelihood of a favorable outcome.

CI, confidence interval; GCS, Glasgow Coma Scale; OR, odds ratio; ROSC, return of spontaneous circulation.

### Feature-specific risk patterns revealed by partial dependence analysis

**Fig. 5:**
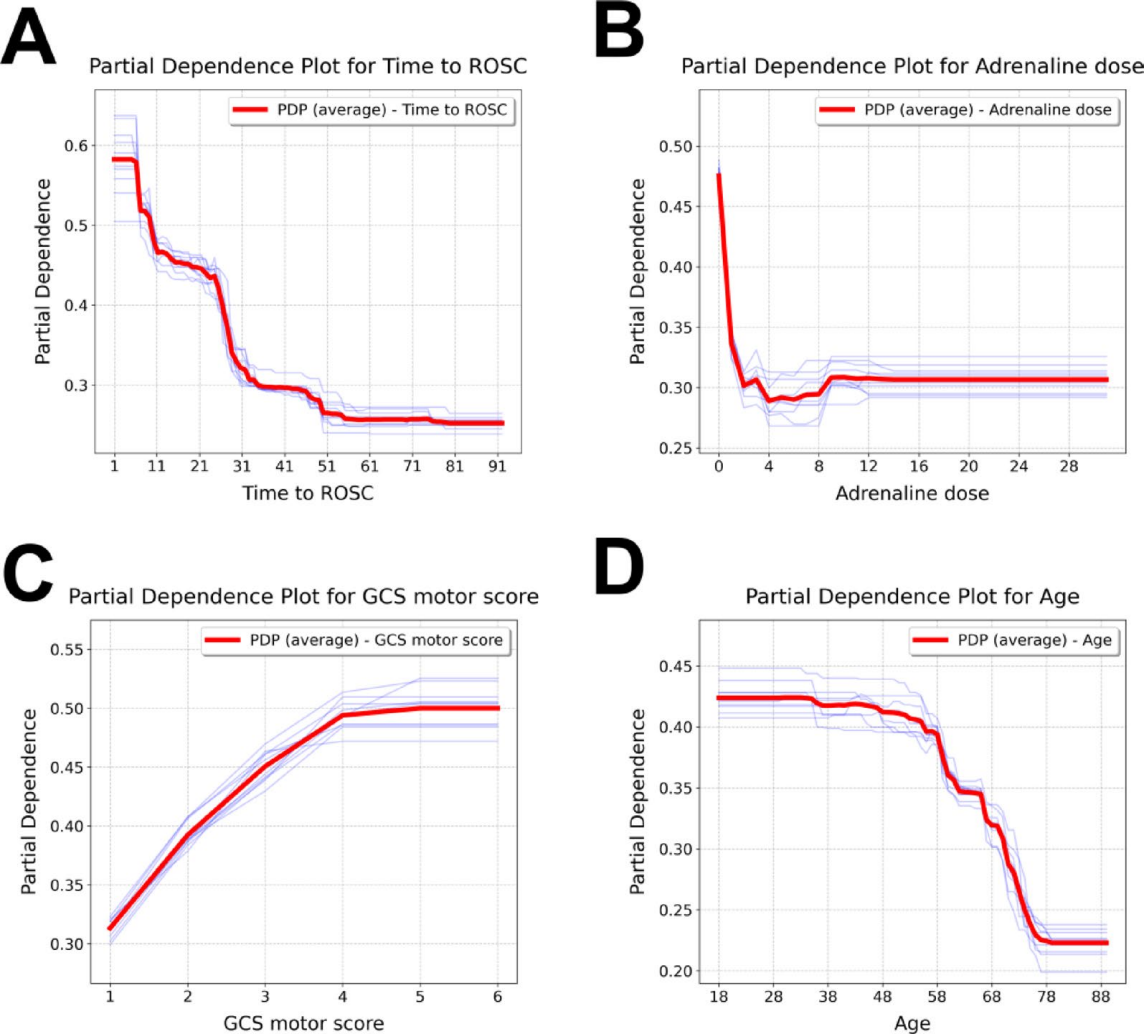
Partial dependence plots (PDPs) of top-ranked continuous predictors in the XGBoost model. This figure depicts the marginal effects of four continuous variables on the predicted probability of favorable neurological outcomes following TTM. In each plot, the bold red line represents the mean PDP across all iterations, while the thin blue lines depict PDPs derived from individual cross-validation folds. **A** time to ROSC exhibited the most substantial impact, with predicted probabilities declining precipitously within the initial 20 min and reaching an asymptotic minimum after 50 min. **B** adrenaline administration demonstrated a complex non-linear relationship: maximal probabilities were observed with minimal or no administration (0–1 mg), followed by a marked decline between 1–4 mg, modest recovery at 5–9 mg, and stabilization beyond 10 mg. **C** GCS motor score showed a positive, approximately linear association with favorable outcomes, particularly from scores 1 to 4, with diminishing incremental benefit beyond a score of 5. **D** age displayed a progressive inverse relationship with predicted outcomes, characterized by a consistent decline after 60 years and minimal further decrease beyond 75 years. Abbreviations: GCS, Glasgow Coma Scale; PDP, partial dependence plot; ROSC, return of spontaneous circulation; TTM, targeted temperature management; XGBoost, eXtreme Gradient Boosting

PDPs from the XGBoost model were used to explore the impact of continuous variables on predictions. Time to ROSC had the steepest risk gradient, with the probability of a favorable outcome declining sharply within 20 min and plateauing beyond 50 min (Fig. 5A). Adrenaline dosage displayed a non-linear relationship, with optimal outcomes at minimal administration (0–1 mg) and a slight rebound before stabilizing at higher doses (Fig. 5B). GCS motor score had a near-linear positive association, with maximal benefit observed between scores 1 and 4 and little added benefit beyond score 5 (Fig. 5C). Age was inversely related to recovery, characterized by progressive probability decrements after age 60 and plateauing at reduced probability levels after age 75 (Fig. 5D).

### Interpreting feature synergies through SHAP interaction heatmaps

**Fig. 6:**
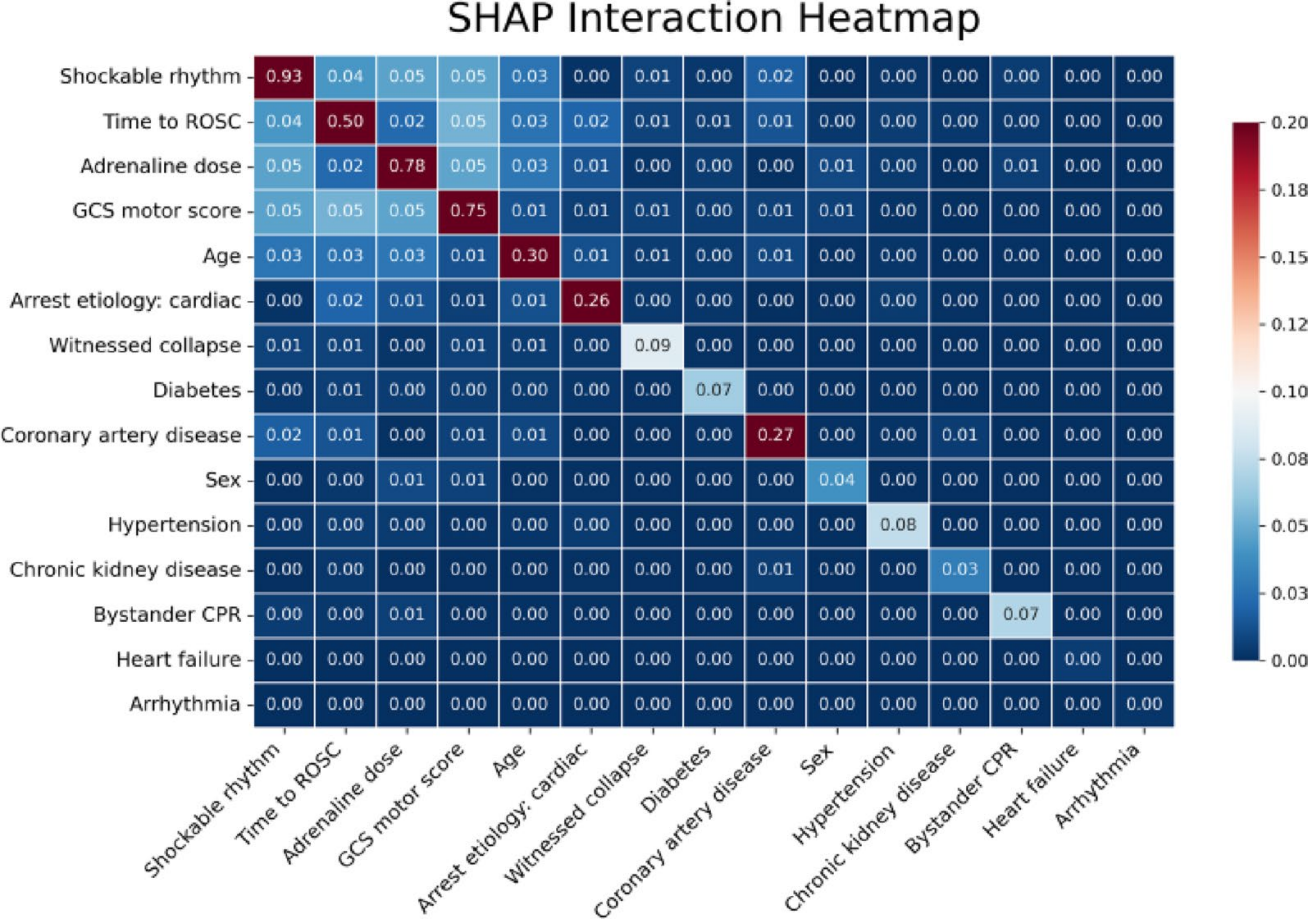
SHAP interaction heatmap of predictive features in the XGBoost model. This heatmap visualizes pairwise SHAP interaction values among the 15 most influential predictors, with stronger interactions indicated by more intense red hues and weaker interactions by blue hues. Notable interactions include shockable rhythm with GCS motor score (0.05), adrenaline dose (0.05), and time to ROSC (0.04), suggesting synergistic effects between initial rhythm, resuscitation interventions, and early neurological responsiveness. Time to ROSC also demonstrated observable interactions with GCS motor score (0.05), age (0.03), and adrenaline dose (0.02), indicating that its prognostic influence may depend on neurological responsiveness and patient-specific factors. Additional relevant interactions were observed between cardiac etiology and ROSC time (0.02) and between coronary artery disease and shockable rhythm (0.02), highlighting context-specific variations. Color intensity was normalized between 0 and 0.2 to enhance visualization; interaction values exceeding 0.2 were clipped for display purposes. Numerical annotations represent actual interaction values. Diagonal elements represent the overall contribution of each feature, including both main effects and self-interaction effects. Abbreviations: CPR, cardiopulmonary resuscitation; GCS, Glasgow Coma Scale; ROSC, return of spontaneous circulation; SHAP, Shapley Additive exPlanations

The SHAP interaction heatmap revealed distinct interdependencies among critical predictors (Fig. 6). Initial shockable rhythm demonstrated prominent interactions with time to ROSC, adrenaline dosage, and GCS motor score, indicating synergistic relationships between cardiac rhythm, resuscitation dynamics, and early neurological responsiveness. Time to ROSC exhibited observable interactions with GCS motor score and adrenaline dosage. Age demonstrated moderate interactions across multiple variables, including shockable rhythm, time to ROSC, and adrenaline dosage, reflecting the complex interplay between baseline patient factors and post-arrest recovery. These patterns indicate that the model integrates individual predictor effects and synergistic interactions, offering insight into the heterogeneous physiology of post-arrest outcomes.

### Patient-level interpretations in the external validation cohort

**Fig. 7:**
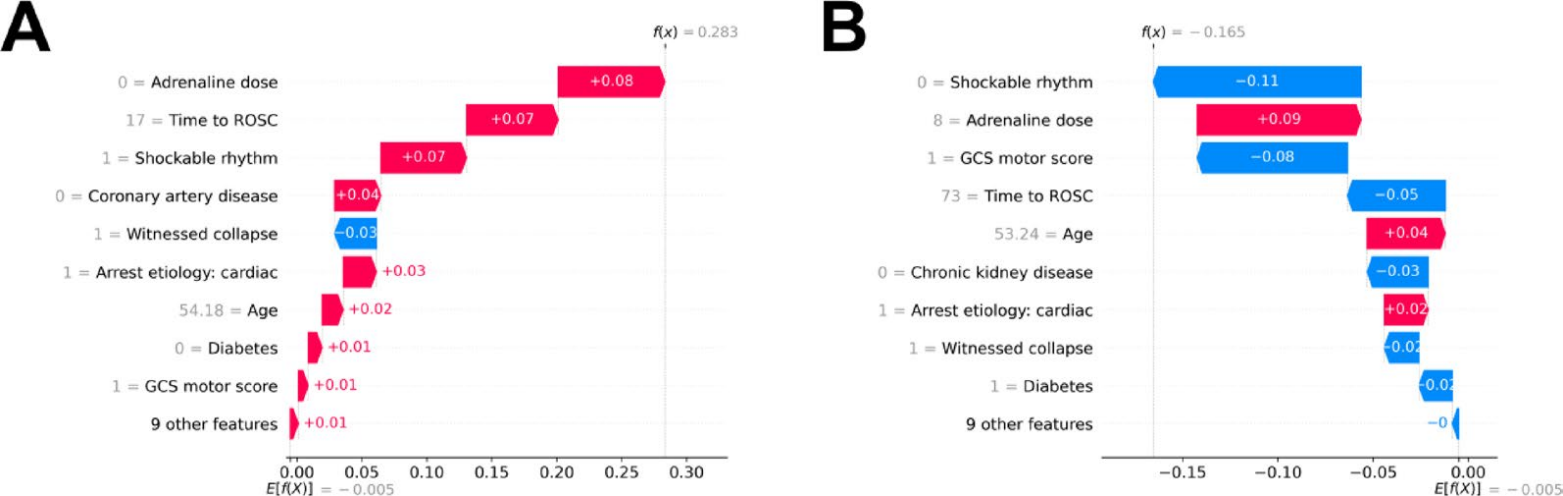
Analysis of patient cases: real-life application of an explainable machine learning model from the external validation cohort.** A** shows a patient with a favorable outcome that the model correctly predicts. The most influential factors included the absence of adrenaline use (+ 0.08), shorter time to ROSC (17 min, + 0.07), shockable rhythm (+ 0.07), and absence of coronary artery disease (+ 0.04). Additional support came from cardiac etiology, moderately young age (54.18 years), and the absence of diabetes. A minor negative effect was outweighed by cumulative positive contributions, aligning with the actual outcome. **B** presents a correctly predicted unfavorable outcome at discharge. The pessimistic prediction was primarily driven by non-shockable rhythm (− 0.11), low GCS motor score (− 0.08), and prolonged time to ROSC (73 min, − 0.05). Although some features, such as adrenaline use (+ 0.09), younger age (+ 0.04), and cardiac etiology (+ 0.02), provided modest positive influence, they were insufficient to outweigh the cumulative negative contributions

Two representative cases were selected from the external validation cohort through stratified random sampling. SHAP waterfall plots were employed to visualize individual predictor contributions, simulating real-world decision-making scenarios. Case 1, who achieved a favorable neurological outcome at discharge, was predicted accurately based on multiple contributing factors, including the absence of adrenaline administration, shorter time to ROSC, shockable initial rhythm, and the absence of coronary artery disease (Fig. 7A). Case 2, with an unfavorable outcome, was also correctly classified. Despite potentially favorable features such as moderate adrenaline dosage, younger age, and cardiac etiology, the prediction was predominantly influenced by adverse factors, including non-shockable rhythm, low GCS motor score, and prolonged time to ROSC (Fig. 7B). These case-level visualizations demonstrate how the model integrates heterogeneous clinical features to generate individualized probability estimates.

## Discussion

This study developed and externally validated an explainable machine learning model for early neurological risk stratification in comatose survivors of OHCA prior to TTM initiation. The model facilitates preliminary patient assessment by identifying individuals at differential risk for neurological outcomes based on data available shortly after ROSC. In the context of evolving post-cardiac arrest care practices, the model is designed as an adjunctive tool to complement comprehensive multimodal evaluation strategies rather than as a replacement for established assessment paradigms. Leveraging two national registries from South Korea (KORHN-pro) and Taiwan (TIMECARD), the model demonstrates robust discriminatory performance across development and external validation cohorts. Cross-validation within the KORHN-pro cohort yielded a mean AUC of 0.925, while external validation using the independent TIMECARD cohort demonstrated an AUC of 0.852, suggesting potential applicability across diverse healthcare systems and patient populations.

A notable methodological strength of this investigation was the integration of contemporary interpretability techniques to enhance algorithm transparency and clinical utility. The model identified clinically significant predictors by applying SHAP and PDP and characterized intertwined relationships and variable interactions. These findings align with established pathophysiological mechanisms of post-cardiac arrest cerebral injury and address limitations of conventional regression-based models, which frequently overlook interdependencies among predictors.

### Decoding outcome complexity via variable interactions

Among top-ranked predictors, five variables—initial cardiac rhythm, time to ROSC, adrenaline dosage, GCS motor score, and age—demonstrated predominant influences on neurological outcomes.

Shockable rhythm emerged as the most potent predictor, with ventricular fibrillation or ventricular tachycardia exhibiting markedly improved prognoses [7, 8, 34]. Its predictive strength was amplified with shorter ROSC times and higher GCS motor scores, highlighting synergy between rapid defibrillation and early neurological responsiveness.

Time to ROSC exhibited steep threshold-dependent patterns, with recovery probabilities declining sharply after 20 min. SHAP interaction analyses demonstrated how it modulated GCS motor score and adrenaline dosage, supporting its status as a key resuscitation metric [16, 35, 36].

Adrenaline demonstrated a dose-dependent inverse relationship with outcomes, with cumulative doses exceeding 5 mg associated with diminished recovery, potentially reflecting arrest severity and adverse adrenergic effects [19, 37, 38]. Its influence varied by age and neurological status, underscoring context-dependent effects.

GCS motor score correlated positively with outcomes, particularly between scores of 1–4, beyond which gains plateaued. Its combined effects with ROSC time and adrenaline dosage validated its integrative role [12, 17, 39].

Age demonstrated a consistent inverse association with favorable outcomes, particularly beyond 75 years. Although less interactive, it remained stable across strata, supporting inclusion in individualized, age-sensitive risk estimation [36, 39, 40].

Results from traditional analyses (Tables 1, 2 and 3; Fig. 4) broadly mirrored the patterns identified by the machine learning model. Although the time to ROSC lost statistical significance in multivariable regression, the overall XGBoost model maintained strong validation performance, and local SHAP explanations illustrated the variable’s continued influence in individual cases. This divergence underscores how machine learning and traditional regression capture different aspects of variable contribution.

These predictors also align with established mechanisms of post-arrest injury and TTM’s neuroprotective effects. Shockable rhythms reflect arrhythmic substrates amenable to rapid defibrillation, shortening ischemic time and limiting neuronal damage [1, 2, 8, 9, 41]. Shorter ROSC time mitigates ischemia-reperfusion cascades—excitotoxicity, oxidative stress, inflammation, and edema—that TTM seeks to counteract [4, 25, 42]. Higher cumulative adrenaline requirements signal refractory arrest physiology. While adrenaline facilitates ROSC, its vasoconstrictive and metabolic effects may impair microcirculation and exacerbate reperfusion injury, potentially explaining its association with worse outcomes [37, 38]. Preserved motor responses indicate residual brainstem-cortical integrity and greater biological reserve for hypothermia-mediated neuroprotection [12, 25, 43]. Advanced age diminishes neuroplasticity and increases the burden of frailty and comorbidities, thereby attenuating the protective impact of TTM.

The physiologic rationale behind these features reinforces the biological plausibility of the model’s predictions [1, 2].

### Contextualizing risk stratification approaches

Traditional risk scores such as OHCA and CAHP show good discrimination in general OHCA populations (C-statistics 0.83–0.84) [10, 22], while their applicability to TTM-treated patients is limited. This subgroup represents survivors with distinct physiological responses and greater recovery potential [5–9], making prognostication more challenging.

Machine learning offers a means to refine prognostication by modeling non-linear, context-dependent relationships [13–15, 19]. Our framework explicitly targets the TTM patients using pre-intervention parameters to estimate recovery potential dynamically. Beyond accuracy, it emphasizes interpretability through feature attribution and interaction analyses, linking algorithmic outputs to physiological mechanisms.

However, model generalizability across healthcare systems remains uncertain. Differences in healthcare resources, including emergency medical services, intensive care capacity, and institutional protocols, as well as regulatory environments, may influence model calibration and performance, while population heterogeneity in comorbidities, socioeconomic, and genetic backgrounds could alter risk distributions and treatment responsiveness. We partially addressed these limitations by harmonizing predictors across two national registries and validating performance in geographically distinct cohorts, addressing the common lack of external validation in prior critical care AI models [13, 14]. Broader validation across diverse healthcare systems and populations will be necessary to determine applicability.

These findings should also be viewed in light of recent trials, particularly TTM2, which showed no significant difference between hypothermia at 33 °C and normothermia [4]. Rather than evaluating temperature strategies, our study developed an explainable machine learning model for patients undergoing TTM treatment. In this regard, our work complements TTM2 by underscoring baseline characteristics—initial rhythm, ROSC time, adrenaline dose, and neurological status—as dominant prognostic determinants. Recent meta-analyses further suggest that hypothermia may benefit subgroups with shockable rhythms or early initiation [8, 9], consistent with our identified predictors.

By delivering individualized, probability-based estimates with interpretability, our model supplements existing tools and extends prognostic precision for TTM-treated OHCA patients, who represent a historically complex yet critical population for targeted neuroprotective interventions.

### Integrative role of machine learning in post-resuscitation assessment

Explainable machine learning, when transparently developed and externally validated, can augment early post-cardiac arrest assessment by translating multidimensional data into individualized insights [44, 45]. The proposed model relies solely on routinely available ROSC variables and simple imputation, enabling seamless integration into clinical workflows as a complementary tool without requiring technical expertise from users.

In exploratory stratification, the model identified a subgroup of TTM patients with a < 5% predicted probability of favorable recovery, among whom actual favorable outcomes were scarce (NPV: 98.9%). These estimates may help contextualize clinical expectations, facilitating informed resource allocation and effective family communication.

Feature importance analysis further suggested that shockable rhythm and ROSC time exert distinct predictive effects under hypothermic conditions, potentially reflecting altered physiological resilience during cooling. These individualized predictions may inform refinement of TTM protocols, such as tailoring rewarming rates or target duration based on patient-specific risk profiles.

Beyond descriptive applications, explainable models may also serve as hypothesis-generating tools. By delineating subgroups with distinct recovery trajectories, they could inform trial design, guide studies on therapeutic responsiveness, and bridge population-level evidence with individualized patient care [45, 46].

## Limitations

This study has several limitations. First, the dataset was derived from Korean and Taiwanese registries, representing predominantly East Asian populations, which may limit generalizability to healthcare systems with different clinical practices, resource availability, or population characteristics. Additionally, subtle differences in inclusion and exclusion criteria between the two registries may have introduced residual selection bias and inter-cohort heterogeneity.

Second, outcomes were limited to neurological status at hospital discharge without long-term functional assessments. This short-term endpoint may limit conclusions regarding sustained recovery.

Third, limited documentation of WLST in the TIMECARD registry may introduce bias related to end-of-life decision patterns, affecting outcome classification. Cultural and legal differences between cohorts further constrain direct comparability.

Fourth, model development was restricted to features consistently available across datasets, excluding additional prognostic modalities such as neuroimaging, biomarkers, or electrophysiological studies that may enhance predictive granularity.

Fifth, analysis was restricted to TTM-treated OHCA patients, as this intervention was consistently applied across registries during the study period. While ensuring population homogeneity and targeting a therapeutically important subgroup, this limits generalizability to patients managed without TTM.

Lastly, the retrospective design using registry data may have introduced selection bias, and despite rigorous adjustment, residual confounding from unmeasured variables cannot be excluded. Moreover, center-specific treatment protocols and institutional practice differences may have influenced patient outcomes. Prospective validation in diverse, multinational cohorts is necessary to establish clinical utility and real-world impact.

## Conclusion

This study developed an interpretable machine learning model for early neurological stratification in comatose survivors of OHCA undergoing TTM. Using harmonized data from two national registries, the model demonstrated consistent performance in internal and external validation cohorts from distinct healthcare systems.

By capturing interactions among critical variables, including initial rhythm, time to ROSC, adrenaline dose, GCS motor score, and age, the model may provide physiologically plausible insights into the determinants of recovery. These findings could assist in early clinical decision-making and refine individualized interpretation of patient trajectories. Identifying synergistic patterns among predictors facilitates hypothesis generation and offers clues to post-arrest pathophysiology, informing future therapeutic considerations.

This explainable framework represents a potential adjunct to conventional clinical assessment in the early post-resuscitation period. By delivering personalized, probability-based estimates, the model is aligned with the principles of precision medicine and may inform more tailored therapeutic and rehabilitation strategies. Further prospective evaluation is warranted to determine its feasibility for integration with real-time monitoring and dynamic risk assessment in critical care.

## Supplementary Information

Supplementary Material 1.

## Data Availability

Data from the Korean Hypothermia Network prospective (KORHN-pro) registry are publicly available and can be accessed at https://doi.org/10.1371/journal.pone.0265275. A de-identified, aggregated version of the Taiwan TIMECARD dataset that supports the findings of this study is available from the corresponding author upon reasonable request, subject to approval by the Research Ethics Review Committee. The analytical code is available from the corresponding author upon reasonable request for non-commercial academic use.
