# Supplementary material for: Explainable machine learning for neurological outcome prediction in out-of-hospital cardiac arrest survivors undergoing targeted temperature management: a multi-cohort validation study
Source: J Neuroeng Rehabil. 2025 Dec 1;23:4. doi: 10.1186/s12984-025-01821-7 (PMC12771708; doi:10.1186/s12984-025-01821-7)
Supplement: Supplementary file 1 — Supplementary Material 1. [file 12984_2025_1821_MOESM1_ESM.pdf]

Supplementary Table 1. TRIPOD-AI checklist

Section/Topic	Item	Development / evaluation[1]	Checklist item	Reported on page
**TITLE**				
Title	1	D;E	Identify the study as developing or evaluating the performance of a multivariable prediction model, the target population, and the outcome to be predicted	**1**
**ABSTRACT**				
Abstract	2	D;E	See TRIPOD+AI for Abstracts checklist	**2**
**INTRODUCTION**				
Background	3a	D;E	Explain the healthcare context (including whether diagnostic or prognostic) and rationale for developing or evaluating the prediction model, including references to existing models	**4**
	3b	D;E	Describe the target population and the intended purpose of the prediction model in the context of the care pathway, including its intended users (e.g., healthcare professionals, patients, public)	**4**
	3c	D;E	Describe any known health inequalities between sociodemographic groups	**4**
Objectives	4	D;E	Specify the study objectives, including whether the study describes the development or validation of a prediction model (or both)	**4**
**METHODS**				
Data	5a	D;E	Describe the sources of data separately for the development and evaluation datasets (e.g., randomised trial, cohort, routine care or registry data), the rationale for using these data, and representativeness of the data	**4–5**
	5b	D;E	Specify the dates of the collected participant data, including start and end of participant accrual; and, if applicable, end of follow-up	**5**
Participants	6a	D;E	Specify key elements of the study setting (e.g., primary care, secondary care, general population) including the number and location of centres	**5**
	6b	D;E	Describe the eligibility criteria for study participants	**5–6**
	6c	D;E	Give details of any treatments received, and how they were handled during model development or evaluation, if relevant	**5**
Data preparation	7	D;E	Describe any data pre-processing and quality checking, including whether this was similar across relevant sociodemographic groups	**7**
Outcome	8a	D;E	Clearly define the outcome that is being predicted and the time horizon, including how and when assessed, the rationale for choosing this outcome, and whether the method of outcome assessment is consistent across sociodemographic groups	**6–7**
	8b	D;E	If outcome assessment requires subjective interpretation, describe the qualifications and demographic characteristics of the outcome assessors	**6**
	8c	D;E	Report any actions to blind assessment of the outcome to be predicted	**N/A**
Predictors	9a	D	Describe the choice of initial predictors (e.g., literature, previous models, all available predictors) and any pre-selection of predictors before model building	**7**
	9b	D;E	Clearly define all predictors, including how and when they were measured (and any actions to blind assessment of predictors for the outcome and other predictors)	**7**
	9c	D;E	If predictor measurement requires subjective interpretation, describe the qualifications and demographic characteristics of the predictor assessors	**7**
Sample size	10	D;E	Explain how the study size was arrived at (separately for development and evaluation), and justify that the study size was sufficient to answer the research question. Include details of any sample size calculation	**9**
Missing data	11	D;E	Describe how missing data were handled. Provide reasons for omitting any data	**7**
Analytical methods	12a	D	Describe how the data were used (e.g., for development and evaluation of model performance) in the analysis, including whether the data were partitioned, considering any sample size requirements	**9–10**
	12b	D	Depending on the type of model, describe how predictors were handled in the analyses (functional form, rescaling, transformation, or any standardisation).	**7**
	12c	D	Specify the type of model, rationale[2], all model-building steps, including any hyperparameter tuning, and method for internal validation	**7–8**
	12d	D;E	Describe if and how any heterogeneity in estimates of model parameter values and model performance was handled and quantified across clusters (e.g., hospitals, countries). See TRIPOD-Cluster for additional considerations[3]	**9–10**
	12e	D;E	Specify all measures and plots used (and their rationale) to evaluate model performance (e.g., discrimination, calibration, clinical utility) and, if relevant, to compare multiple models	**10–12,**
	12f	E	Describe any model updating (e.g., recalibration) arising from the model evaluation, either overall or for particular sociodemographic groups or settings	**N/A**
	12g	E	For model evaluation, describe how the model predictions were calculated (e.g., formula, code, object, application programming interface)	**7–8**
Class imbalance	13	D;E	If class imbalance methods were used, state why and how this was done, and any subsequent methods to recalibrate the model or the model predictions	**7–8**
Fairness	14	D;E	Describe any approaches that were used to address model fairness and their rationale	**N/A**
Model output	15	D	Specify the output of the prediction model (e.g., probabilities, classification). Provide details and rationale for any classification and how the thresholds were identified	**7–8**

[1] D=items relevant only to the development of a prediction model; E=items relating solely to the evaluation of a prediction model; D;E=items applicable to both the development and evaluation of a prediction model
[2] Separately for all model building approaches.
[3] TRIPOD-Cluster is a checklist of reporting recommendations for studies developing or validating models that explicitly account for clustering or explore heterogeneity in model performance (eg, at different hospitals or centres). Debray et al, BMJ 2023; 380: e071018 [DOI: 10.1136/bmj-2022-071018]

Training versus evaluation	16	D;E	Identify any differences between the development and evaluation data in healthcare setting, eligibility criteria, outcome, and predictors	**5**
Ethical approval	17	D;E	Name the institutional research board or ethics committee that approved the study and describe the participant-informed consent or the ethics committee waiver of informed consent	**20**
**OPEN SCIENCE**				
Funding	18a	D;E	Give the source of funding and the role of the funders for the present study	**20**
Conflicts of interest	18b	D;E	Declare any conflicts of interest and financial disclosures for all authors	**20**
Protocol	18c	D;E	Indicate where the study protocol can be accessed or state that a protocol was not prepared	**N/A**
Registration	18d	D;E	Provide registration information for the study, including register name and registration number, or state that the study was not registered	**N/A**
Data sharing	18e	D;E	Provide details of the availability of the study data	**20**
Code sharing	18f	D;E	Provide details of the availability of the analytical code[4]	**20**
**PATIENT & PUBLIC INVOLVEMENT**				
Patient & Public Involvement	19	D;E	Provide details of any patient and public involvement during the design, conduct, reporting, interpretation, or dissemination of the study or state no involvement.	**N/A**
**RESULTS**				
Participants	20a	D;E	Describe the flow of participants through the study, including the number of participants with and without the outcome and, if applicable, a summary of the follow-up time. A diagram may be helpful.	**9–10**
	20b	D;E	Report the characteristics overall and, where applicable, for each data source or setting, including the key dates, key predictors (including demographics), treatments received, sample size, number of outcome events, follow-up time, and amount of missing data. A table may be helpful. Report any differences across key demographic groups.	**9–10**
	20c	E	For model evaluation, show a comparison with the development data of the distribution of important predictors (demographics, predictors, and outcome).	**9–10**
Model development	21	D;E	Specify the number of participants and outcome events in each analysis (e.g., for model development, hyperparameter tuning, model evaluation)	**9–10**
Model specification	22	D	Provide details of the full prediction model (e.g., formula, code, object, application programming interface) to allow predictions in new individuals and to enable third-party evaluation and implementation, including any restrictions to access or re-use (e.g., freely available, proprietary)[5]	**7–8, 20**
Model performance	23a	D;E	Report model performance estimates with confidence intervals, including for any key subgroups (e.g., sociodemographic). Consider plots to aid presentation.	**10–11**
	23b	D;E	If examined, report results of any heterogeneity in model performance across clusters. See TRIPOD Cluster for additional details[3].	**10–12**
Model updating	24	E	Report the results from any model updating, including the updated model and subsequent performance	**N/A**
**DISCUSSION**				
Interpretation	25	D;E	Give an overall interpretation of the main results, including issues of fairness in the context of the objectives and previous studies	**12–15**
Limitations	26	D;E	Discuss any limitations of the study (such as a non-representative sample, sample size, overfitting, missing data) and their effects on any biases, statistical uncertainty, and generalizability	**15–16**
Usability of the model in the context of current care	27a	D	Describe how poor quality or unavailable input data (e.g., predictor values) should be assessed and handled when implementing the prediction model	**7**
	27b	D	Specify whether users will be required to interact in the handling of the input data or use of the model, and what level of expertise is required of users	**14–15**
	27c	D;E	Discuss any next steps for future research, with a specific view to applicability and generalizability of the model	**15**

From: Collins GS, Moons KGM, Dhiman P, et al. *BMJ* 2024;385:e078378. doi:10.1136/bmj-2023-078378

[4] This relates to the analysis code, for example, any data cleaning, feature engineering, model building, evaluation.
[5] This relates to the code to implement the model to get estimates of risk for a new individual.

TRIPOD-AI Checklist with Item-Level Explanations of Non-Applicable Items

Item 8c – Blinding of Outcome Assessment
N/A. This study utilized retrospectively collected data from two national cardiac arrest registries. Neurological outcomes (CPC scores) were recorded as part of routine clinical documentation by the treating physicians. No additional blinding procedures were implemented, and such procedures were not feasible, given the nature of the data sources.

Item 12f – Model Updating
N/A. No model updating, recalibration, or refitting was performed. External validation was conducted using fixed models trained on the development cohort, with the classification threshold determined during internal validation. This approach was intended to reflect real-world deployment without data leakage or tuning on the external dataset.

Item 14 – Model Fairness
N/A. No specific fairness evaluation was conducted, as this study utilized de-identified registry data with limited sociodemographic granularity. The registries did not collect detailed information on ethnicity or socioeconomic status, precluding subgroup fairness analysis.

Item 18c – Study Protocol
N/A. No pre-specified study protocol was registered or made publicly available for this secondary analysis. All analyses were conducted based on a predefined internal plan prior to model development.

Item 18d – Study Registration
N/A. This retrospective modeling study was not prospectively registered in a public registry.

Item 19 – Patient and Public Involvement
N/A. There was no direct involvement of patients or members of the public in the design, conduct, analysis, or reporting of this retrospective registry-based study.

Item 24 – Model Updating
N/A. No model updating or recalibration was performed following external validation. The XGBoost model was applied to the external validation cohort without retraining or threshold adjustment, as described in the Methods section.

Supplementary Table 2. Predictive Performance of Machine Learning Models for Neurological Outcomes in the Development Cohort

Model	AUC	Accuracy	Precision	Recall	Specificity
Logistic Regression	0.909 (0.89–0.93)	0.859 (0.83–0.88)	0.796 (0.72–0.87)	0.794 (0.73–0.86)	0.890 (0.84–0.94)
SVM	0.908 (0.89–0.93)	0.851 (0.83–0.87)	0.792 (0.74–0.84)	0.741 (0.69–0.79)	0.904 (0.88–0.93)
Random Forest	0.920 (0.90–0.94)	0.856 (0.83–0.88	0.777 (0.71–0.85)	0.809 (0.76–0.86)	0.879 (0.83–0.93)
Extra Trees	0.914 (0.90–0.93)	0.853 (0.83–0.88)	0.761 (0.70–0.83)	0.829 (0.78–0.88	0.865 (0.81–0.92)
LightGBM	0.917 (0.90–0.93)	**0.868** (0.85–0.88)	0.812 (0.77–0.86)	0.785 (0.72–0.85)	0.907 (0.87–0.94)
ANN	0.906 (0.88–0.93)	0.857 (0.83–0.89)	**0.819** (0.75–0.89)	0.738 (0.70–0.77)	**0.914** (0.87–0.96
XGBoost	**0.925** (0.91–0.94)	0.863 (0.84–0.89)	0.770 (0.72–0.82)	**0.835** (0.81–0.86)	0.876 (0.84–0.91)

Notes: The highest value of the predictive metric among the machine learning models is shown in boldface. Data in parentheses represent 95% confidence intervals (CIs).

Abbreviations: ANN, artificial neural network; AUC, area under the receiver operating characteristic curve; Extra Trees, extremely randomized trees; LightGBM, light gradient boosting machine; SVM, support vector machine; XGBoost, eXtreme gradient boosting.

Supplementary Table 3. Predictive Performance of Machine Learning Models for Neurological Outcomes in the External Validation Cohort

Model	AUC	Accuracy	Precision	Recall	Specificity
Logistic Regression	0.808 (0.80–0.81)	0.781 (0.78–0.79)	0.524 (0.52–0.53)	0.608 (0.59–0.62)	0.833 (0.83–0.84)
SVM	0.808 (0.80–0.81)	0.813 (0.81–0.82)	0.601 (0.60–0.62)	0.533 (0.53–0.54)	0.897 (0.89–0.90)
Random Forest	0.840 (0.84–0.84)	0.819 (0.81–0.82)	0.594 (0.58–0.61)	0.690 (0.69–0.70)	0.857 (0.85–0.87)
Extra Trees	0.843 (0.84–0.84)	0.806 (0.80–0.81)	0.576 (0.56–0.59)	0.629 (0.62–0.64)	0.860 (0.85–0.87)
LightGBM	0.844 (0.84–0.85)	0.792 (0.78–0.80)	0.539 (0.53–0.55)	**0.724** (0.71–0.74)	0.813 (0.80–0.82)
ANN	0.821 (0.82–0.82)	**0.820** (0.82–0.82)	**0.627** (0.61–0.64)	0.546 (0.54–0.56)	**0.902** (0.90–0.91)
XGBoost	**0.852** (0.85–0.85)	0.809 (0.80–0.82)	0.584 (0.57–0.60)	0.617 (0.61–0.63)	0.867 (0.86–0.88)

Notes: The highest value of the predictive metric among the machine learning models is shown in boldface.

Data in parentheses represent 95% confidence intervals (CIs).

Abbreviations: ANN, artificial neural network; AUC, area under the receiver operating characteristic curve; Extra Trees, extremely randomized trees; LightGBM, light gradient boosting machine; SVM, support vector machine; XGBoost, eXtreme gradient boosting.

Supplementary Table 4. Final hyperparameter values and search ranges for the XGBoost model

Hyperparameter	Final Value	Search Range	Notes
n_estimators	326	50–1000	Bayesian optimization, 30,000 trials
max_depth	8	3–15	
learning_rate	0.109	0.01–0.3	
min_child_weight	2	1–10	
subsample	0.879	0.5–1.0	
colsample_bytree	0.686	0.5–1.0	
gamma	3.447	0–5	
reg_lambda	1.0	0.0–5.0	L2 regularization
reg_alpha	0	0.0–5.0	L1 regularization
scale_pos_weight	1	1	Fixed at 1; class imbalance handled with SMOTE

Notes: Hyperparameters were optimized via Optuna Bayesian optimization using 10-fold cross-validation. SMOTE was applied during training to balance classes. Search ranges and iteration settings are indicated above.

Abbreviations: SMOTE, Synthetic Minority Oversampling Technique; XGBoost, eXtreme gradient boosting.

Supplementary Figure. Calibration plot of the XGBoost model on the external validation cohort.

Predicted probabilities were grouped into deciles and plotted as a calibration curve (solid line), with the diagonal dashed line representing perfect agreement between predicted and observed event rates. The model demonstrated good calibration, with predicted probabilities closely tracking observed outcomes across the risk spectrum. The Brier score was 0.129, which reflects acceptable overall calibration performance [1, 2].

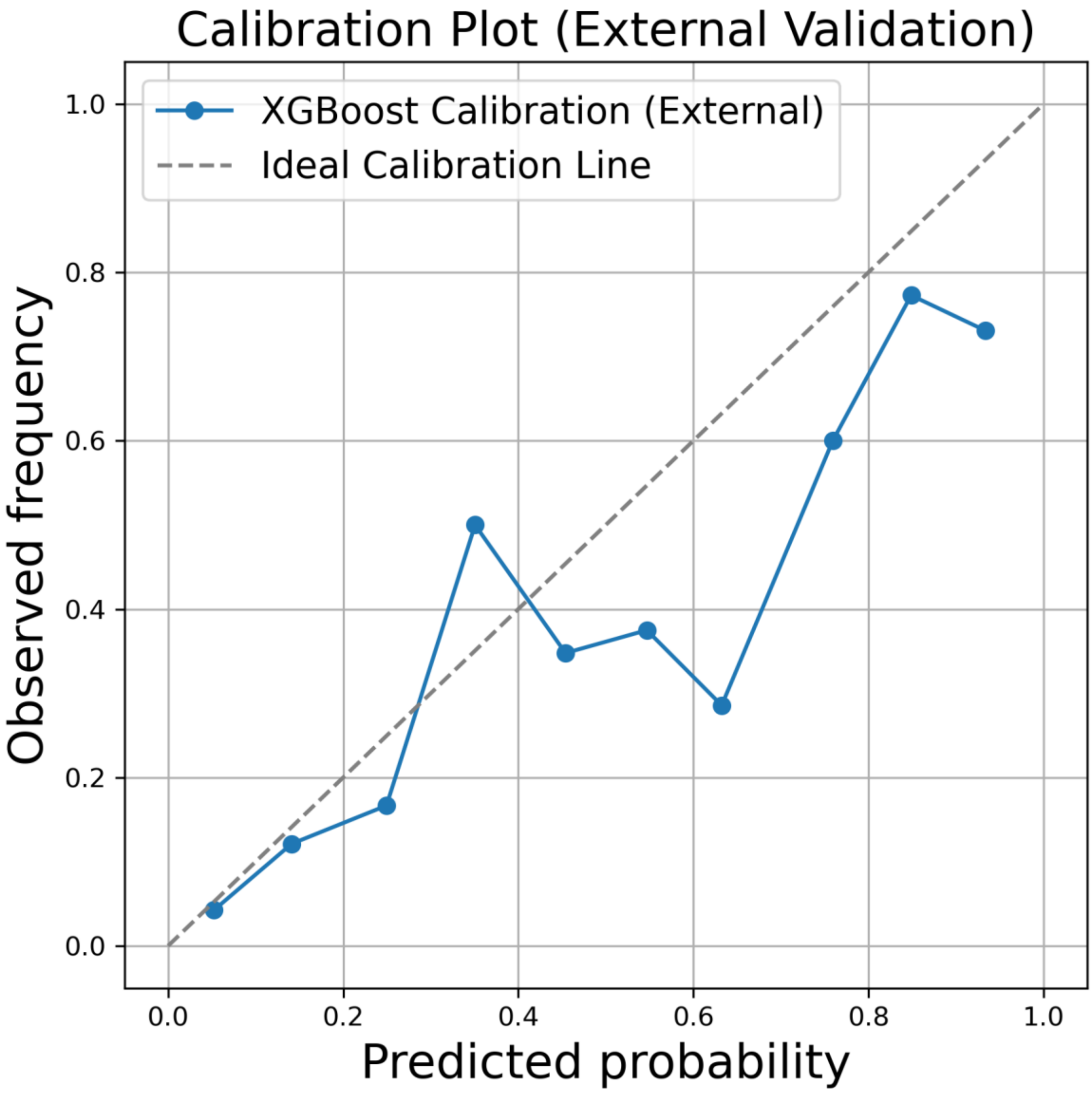

References:

1. Steyerberg EW, Vickers AJ, Cook NR, Gerds T, Gonen M, Obuchowski N, et al. Assessing the performance of prediction models: a framework for traditional and novel measures. Epidemiology. 2010;21(1):128-38. doi: 10.1097/EDE.0b013e3181c30fb2.
2. Steyerberg EW. Clinical prediction models: a practical approach to development, validation, and updating. 1 ed. New York: Springer; 2009.
